# Global health issues of aflatoxins in food and agriculture: challenges and opportunities

**DOI:** 10.3389/fmicb.2014.00420

**Published:** 2014-08-12

**Authors:** Mehdi Razzaghi-Abyaneh, Perng-Kuang Chang, Masoomeh Shams-Ghahfarokhi, Mahendra Rai

**Affiliations:** ^1^Research Laboratory of Fungal Metabolites, Department of Mycology, Pasteur Institute of IranTehran, Iran; ^2^Food and Feed Research Unit, Southern Regional Research CenterNew Orleans, LA, USA; ^3^Department of Mycology, Faculty of Medical Sciences, Tarbiat Modares UniversityTehran, Iran; ^4^Department of Biotechnology, Sant Gadge Baba Amravati UniversityAmravati, India

**Keywords:** aflatoxin, *Aspergillus*, food, agriculture, public health

Aflatoxins are a group of polyketide mycotoxins that are produced mainly by members of the genus *Aspergillus*. Production of these toxic secondary metabolites is closely related to fungal development (Keller et al., 2005; Jamali et al., 2012). Contamination of food, feed and agricultural commodities by aflatoxins poses enormous economic and serious health concerns because these chemicals are highly carcinogenic and can directly influence the structure of DNA. The resulting genetic defects can lead to fetal misdevelopment and miscarriages; aflatoxins are also known to suppress immune systems (Razzaghi-Abyaneh et al., 2013). In a global context, aflatoxin contamination is a constant concern between the 35N and 35S latitude where developing countries are mainly situated. With expanding boundaries of developing countries, aflatoxin contamination has become a persistent problem to those emerging areas (Shams-Ghahfarokhi et al., 2013). The continuing threat by aflatoxin contamination of food, feed and agricultural commodities to the world population has made aflatoxin research one of the most exciting and rapidly developing study areas of microbial toxins.

The present research topic includes six review articles, three mini reviews and four original research articles. Contributors highlight current global health issues arising from aflatoxins and aflatoxigenic fungi and cover important aspects of aflatoxin research including contamination of crops, epidemiology, molecular biology and management strategies. Special attention is given to fungus-plant host interactions, biodiversity and biocontrol, sexual recombination in aflatoxigenic aspergilli, potential biomarkers for aflatoxin exposure in humans and safe storage programs.

Perrone et al. (2014) reported the expected risk of a shift in aflatoxin problems toward new territories particular in South East of Europe due to increasing average temperatures. Giving an overview on genetic diversity of *A*. *flavus* populations in Europe, the authors stressed the importance of selecting stable atoxigenic *A. flavus* strains as biocontrol agents. In the review of climate change on *A. flavus* growth and aflatoxin production, Medina et al. (2014) focused on the potential impact of key environmental factors, such as water activity (a_w_), temperature and atmospheric CO_2_, and their interactions on ecology, growth and aflatoxin production by the *A. flavus* both *in vitro* and on maize. The authors showed that while such interacting abiotic factors have little effect on fungal growth, they however have a significant impact on aflatoxin biosynthetic gene expression and can stimulate the production of aflatoxins. In the insightful mini-review on sexual recombination in aflatoxin-producing *Aspergillus* species, Moore (2014) concisely summarized the potential negative impact of sexual recombination on the feasibility of using biological controls to reduce aflatoxin contamination of field crops. The author discussed specifically the implication of sexual recombination on the fate of two commercially available biopesticides: AF36 and Afla-Guard®. In the excellent review on the characteristics of *A. flavus* as well as the biocontrol strategy using non-toxigenic *A. flavus* strains, Ehrlich (2014) described the current state and outlook of this application in agricultural field. The author concluded that understanding genetic variations among *A. flavus* strains is critical for developing a robust biocontrol strategy, and it is unlikely that a “one size fits all” strategy will work for preharvest aflatoxin reduction.

Host resistance is a very attractive area on aflatoxin research, and various aspects of *A. flavus*-plant host interaction were investigated with special focuses on mechanisms resistant to fungal growth and aflatoxin production (Dolezal et al., 2014; Fountain et al., 2014; Hruska et al., 2014; Scarpari et al., 2014; Shan and Williams, 2014). In the up-to-date review on environmental influences on aflatoxin production on maize, Fountain et al. (2014) detailed the history of research on this complex interaction and pointed out future directions for elucidating host resistance and susceptibility to *A. flavus* colonization in relation to abiotic stress such as drought and heat stresses, and oxidative stress in which aflatoxin may function as an antioxidant to the producing fungus. Utilizing an aflatoxigenic Green Fluorescence Protein (GFP) *A. flavu*s strain, Hruska et al. (2014) investigated invasion, colonization and competition in maize kernels by this engineered strain. The authors showed that a decrease in aflatoxin production is correlated with depression of the aflatoxigenic population by the biocontrol strain, AF36, supporting the theory of competitive exclusion. Using a lipidomic approach to investigate *A. flavus*-maize interactions, Scarpari et al. (2014) suggested that *A. flavus* elicits the production of oxylipins in host plants, which function as signals for regulating aflatoxins biosynthesis, conidiogenesis and sclerotia formation. Their results highlighted the important role of maize oxylipins in driving secondary metabolism in *A. flavus*. In the microarray study to identify maize genes expressed during pathogen infection, Dolezal et al. (2014) found that metabolic processes are linked to defense responses, which include physical changes within the kernel as well as a disruption in kernel development. Shan and Williams (2014) provided a concise but clear overview of current knowledge about quantitative trait loci of corn related to aflatoxin contamination and ongoing efforts in the development of resistant corn lines. The authors concluded that the “phenotypic traits/data” established based on transcriptomics and proteomics approaches could be translated into the practices for improving corn resistance.

Human exposure to aflatoxins is another challenging but not well studied area. In the concise overview of aflatoxin contamination of foods and related biomarker research, Mohd-Redzwan et al. (2013) described the historical problems related to aflatoxins in Malaysia, and how these problems have influenced the Malaysian population by highlighting the aflatoxin concentrations in basic food products and their comparison with established aflatoxin limits. The authors emphasized the importance of the legislation of law for a more controlled food production, legal enforcement to meet the set regulatory standards, and the improvement of pre and post-harvest techniques to reduce aflatoxin amounts in food and hence to decrease diseases in Malaysian population. In the comprehensive review about using microRNAs as specific molecular biomarkers in populations exposed to aflatoxins and as early markers for evidence of presence of or damage by hepatocellular carcinoma (HCC), Valencia-Quintana et al. (2014) described differential expression of microRNAs under specific conditions related not only to chemical and environmental pollutants but also to biological pollutants such as the presence of aflatoxins in humans and animals, and consequently, their influence over HCC. The authors provided findings that are important to toxicological research because microRNAs can be used to predict the toxicity of some compounds and will help to explore new treatments.

In a well designed study aimed at enhancing antimycotic activities of known antifungal chemicals by natural compounds, Kim et al. (2014) successfully increased chemosensitization of a kresoxim methyl (Kre-Me), a natural fungicide from strobilurin class by chemically-synthesized benzo analogs. The authors found that among tested benzo analogs, octylgallate (OG) inhibits both growth and aflatoxin production by toxigenic aspergilli more efficiently. The study provided good evidence of remarkable synergism between OG and Kre-Me, which enhances the effectiveness of Kre-Me considerably. The efficient chemosensitizing capability of OG in increasing the efficacy of Kre-Me could reduce effective dosages of strobilurins and alleviate negative side effects associated with the current antifungal treatment.

Finally, as one of the most important and practical issues on aflatoxin research, aflatoxin prevention and elimination, Villers (2014) presented laboratory and field data on an Ultra Hermetic™ storage system, which creates an unbreatheable atmosphere through insect and microorganism respiration alone, in preventing the exponential production and accumulation of aflatoxins. This system is proven useful during multi-month post-harvest safe storage tests of maize, rice and peanuts in hot, humid countries. The author further stressed the need for research on post-harvest protection against aflatoxin contamination by determining the frequency at which excessive aflatoxin levels are reached in the field vs. after months of post-harvest storage using this system.

In conclusion, this research topic opens exciting perspectives on global health issues related to aflatoxins in the food chain and on the development of suitable strategies for preventing toxigenic fungal growth in field and storage, thereby reducing or eliminating subsequent aflatoxin contamination of our food supplies.

## Conflict of interest statement

The authors declare that the research was conducted in the absence of any commercial or financial relationships that could be construed as a potential conflict of interest.
